# Prostate specific G protein coupled receptor is associated with prostate cancer prognosis and affects cancer cell proliferation and invasion

**DOI:** 10.1186/s12885-015-1921-6

**Published:** 2015-11-18

**Authors:** Wenqing Cao, Faqian Li, Jorge Yao, Jiangzhou Yu

**Affiliations:** 1 Department of Pathology and Laboratory Medicine, University of Rochester Medical Center, Rochester, NY USA; 2 Department of Pathology, New York University Langone Medical Center, New York City, NY USA; 3 Department of Laboratory Medicine and Pathology, University of Minnesota, Minneapolis, MN USA; 4 Pathline Laboratories, Ramsey, NJ USA

**Keywords:** PSGR, Prostate cancer, Expression, Invasion, Prognosis

## Abstract

**Background:**

There is limited information about the clinical and biological significance of prostate specific G protein coupled receptor (PSGR) in prostate cancer (PCa) initiation and progression. Here, we evaluated the expression of PSGR protein, studied its diagnostic and prognostic value in PCa, and also explored its role in cancer cell growth and invasion.

**Methods:**

The expression of PSGR in paired adjacent normal prostate, high grade prostatic intraepithelial neoplasia (PIN), and PCa were determined by immunohistochemistry on tissue microarrays constructed from 150 radical prostatectomy specimens. The effects of PSGR on PCa cell growth and invasion were investigated using human PCa cell lines.

**Results:**

Membranous and cytoplasmic PSGR staining was observed at luminal epithelial cells of prostate. PSGR protein expression was significantly higher in PIN compared to normal prostate. Interestingly, the expression of PSGR decreased as PIN progressed to PCa. Low PSGR expression in PCa was associated with high Gleason score, and poor overall survival. Activated PSGR increased cancer cell invasive ability, but retarded cell growth. PSGR did not affect mTOR activity, but suppressed P70 S6 kinase activity.

**Conclusions:**

PSGR may participate in PCa progression through affecting cell proliferation and invasion. High expression of PSGR in PIN may implicate its role in early neoplastic transformation of PCa. Low expression of PSGR in PCa may serve as a potential indicator for poor prognosis.

## Background

Prostate cancer (PCa) is the most common neoplasia and the second leading cause of death from cancer in American men [1]. To further improve patient outcome, more efforts are needed to study the molecular mechanisms underlying PCa development and progression. Identification of new molecular markers for PCa would allow more reliable diagnosis and prognosis, and provide potential targets for optimizing therapeutic strategies.

Prostate Specific G protein Coupled Receptor (PSGR) is a G protein coupled receptor that has been found to have restricted expression in human prostate tissues by Northern blot and real-time PCR analysis of over 50 different human tissue types [2]. The mRNA level of PSGR increases significantly in the epithelial cells of prostate intraepithelial neoplasia (PIN) and PCa compared to non-cancerous controls and benign prostatic hyperplasia (BPH), suggesting that PSGR may play important roles in prostate cancer development and progression [2–4]. A high mRNA level of PSGR seems to correlate with prostate specific antigen (PSA) level [5] but not with patient age, tumor stage, Gleason score, lymphovascular invasion, or recurrence [3, 5, 6]. PSGR transcripts in urine may be used as an early diagnostic marker for PCa [7]. A previous study with a panel of PSA, prostate cancer gene 3 (PCA3), PSGR and α-methylacyl-CoA racemase (AMACR) indicated that PSGR transcript could increase the diagnostic specificity [6].

Recently, Neuhaus et al. reported that β-ionone, a specific agonist of PSGR, could increase intracellular Ca^2+^ flux [8]. They found that endogenous Ca^2+^-selective transient receptor potential vanilloid type 6 (TRPV6) channels were downstream molecules to mediate the signaling from activated PSGR, which was dependent on Src kinase activity [9]. Moreover, their data showed that activated PSGR inhibited proliferation of LNCaP cells by increasing apoptosis through MAPK signaling [8]. Notably, a recent study from Sanz et al. [10] reported that activation of PSGR with β-ionone promoted migration and invasion of LNCaP cells, as well as metastasis in animal experiments.

These findings give rise to the possibility of using PSGR as a marker for early diagnosis and prognosis, and suggest a novel potential target for PCa treatment [4]. However, the endogenous ligands, biological, and pathological significance of PSGR in the prostate remain unidentified. To date, there has been little evaluation of PSGR protein expression in normal prostate and PCa. Therefore in this study, using immunohistochemistry, we evaluated PSGR protein expression in various normal human tissues, several PCa cell lines, and a cohort of 150 PCa specimens. We further studied the associations of PSGR protein expression in PCa with related clinical parameters and patient overall survival (OS). In addition, we tested how activated PSGR affected PCa cell proliferation and invasion.

## Methods

### Patients’ recruitment and tissue samples

The pathology database at the University of Rochester Medical Center (URMC) from 2004 to 2005 was searched for radical prostatectomy and prostatic adenocarcinoma. 150 cases were identified and retrieved for histologic review. The diagnosis of prostatic adenocarcinoma for each case was confirmed by one of the two pathologists (FL and WQC). Clinical and pathological information including age, stage, Gleason score, lymphovascular invasion, extraprostatic extension, and survival were collected from the clinical notes and pathology reports. Human tissue specimens obtained and processed at URMC were collected under the protocol “Studies of biomarker expression in human tumors” (RSRB00020130) approved by the University of Rochester Research Subjects Review Board (RSRB). The institutional review board RSRB approved this retrospective study and waived the need for consent. Patient records/information was anonymized and de-identified prior to analysis.

### Tissue microarrays

Archival, formalin-fixed, paraffin-embedded tissue blocks from the selected patients were procured from the Department of Pathology and Laboratory Medicine at URMC. Tissue microarrays (TMAs) were constructed from the selected paraffin blocks of 150 cases. For each case, areas containing benign prostatic tissue, high grade prostatic intraepithelial neoplasia, or prostatic adenocarcinoma were first marked by a pathologist for sampling. Two 3 mm core samples were then retrieved from each selected area in the donor paraffin blocks, and transferred to a TMA paraffin block. Six cores were taken from each individual case. A TMA grid map was made to keep track of cores from the same prostatectomy specimen. The Gleason grade for each PCa core was the same as that in the final pathology report. Many tissue cores contained two of the three tissue types (normal and PIN, PIN and PCa). Each TMA block also included tissue cores (normal liver, placenta, thyroid tissue, or small bowel) as controls.

### Reagents

α-ionone and β-ionone were purchased from Sigma (St Louis, MO or NU-chek prep, INC. Elysian, MO), dissolved in DMSO and stored at −80 °C. Specific antibodies against AR, PSGR, Phosphorylated P70 S6 kinase, phosphorylated mTOR, and phosphorylated 4EBP1 were obtained from Abcam (Cambridge, MA). The antibody against PSGR for immunohistochemistry was from Novus Biologicals (Littleton, CO).

### Immunohistochemistry

Tissue microarray sections were immunohistochemically stained with PSGR antibody as previously described [11]. Briefly, TMA sections were de-paraffinized and rehydrated through graded alcohols. Antigen retrieval was performed by boiling in an EDTA buffer (pH 9.0) at 98 °C for 20 min. Endogenous peroxidase activity was blocked with 3 % hydrogen peroxide. The slides were incubated with antibody against PSGR (NLS6332, rabbit polyclonal, 1: 100) at room temperature for one hour and were subsequently incubated for 30 min with EnVision + System horseradish peroxidase-labeled polymer conjugated with biotinylated anti-rabbit secondary antibody and 3,3′-diaminobenzidine substrate. All sections were counterstained with Mayer’s hematoxylin. Normal prostate and testes were used as positive and negative control for PSGR. Negative controls were also established by the replacement of primary antibodies with normal serum.

### Evaluation of immunohistochemical staining

**Fig. 1:**
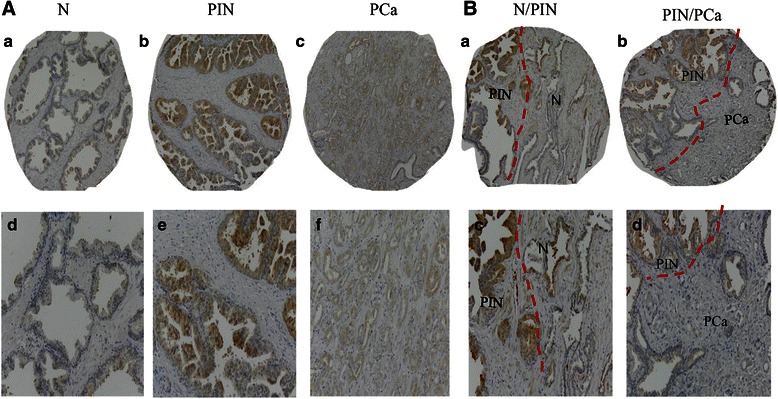
Expression of PSGR protein in normal prostate (N), prostatic intraepithelial neoplasia (PIN) and prostate cancer (PCa) by immunohistochemistry. **A** Low (*a-c*) and high magnification (*d-f*) images of PSGR staining in N (*a*, *d*), PIN (*b, e*) and PCa (*c*, *f*); **B** Low (*a* and *b*) and high (*c* and *d*) magnification images of PSGR in N and adjacent PIN (*a*, *c*), and PIN and adjacent PCa (*b*, *d*) (original magnification x 10 and x 20 for low and high respectively)

Membranous and cytoplasmic PSGR staining was observed in the epithelial cell of normal prostate, PIN and PCa (Fig. 1a). To more precisely represent the expression level of PSGR, the H-score system was employed in this study. Two independent researchers (JZY and WQC) evaluated the stained slides as described previously [12]. The staining intensity for each protein was scored as 0, no staining; 1+, weak; 2+, moderate; and 3+, strong staining. The percentage of tumor cells that stained positive was also estimated (0–100 %). A H score (range 0–300) was generated by multiplying the staining intensity score and the percentage of positively stained tumor cells (H-Score = 3× percentage of cells with strong staining + 2× percentage of cells with moderate staining + 1× percentage of cells with weak staining + 0× percentage of cells with no staining). The Kappa value for inter-observer agreement between the two researchers was 0.85.

### Cell culture and invasion assay

LNCaP, PC3, and C4-2 human PCa cell lines were obtained from the American Type Culture Collection (ATCC). Cells were cultured in Dulbecco’s modified Eagle’s medium, supplemented with 10 % of FBS and antibiotics in 100 mm cell culture dishes or culture plates. 12 h prior to experimentation, cells were cultured in Dulbecco’s modified Eagle’s medium with 5 % charcoal treated FBS and antibiotics.

To measure cell invasion, an insert with 8.0 μm pores (Corning) was coated with matrigel (BD Biosciences) and 5 × 10^5^ C4-2 cells were seeded. After 3 days with β- or α-ionone treatment, the cells remaining in the upper compartment of insert were removed by cotton swabs, and those invaded through the matrix were stained with 0.1 % crystal violet and counted under a light microscope at five individual fields per insert. Results were presented as an average of triplicate experiments.

### Cell proliferation and growth assay

MTT (3-(4,5-dimethylthiazol-2-yl)-2,5- diphenyltetrazolium bromide) assay and anchorage-independent growth assay were performed to measure cell proliferation following previously published protocols [13]. Briefly, for MTT assay, cells were seeded in 24-well plates. After 3 days culture, the medium was removed and the cells were washed once with PBS. 300 μl of serum-free medium and 30 μl of MTT reagent (5 mg/ml) were added, and incubated for one and a half hours in a humidified 5 % CO2 incubator at 37 °C. The absorbance was measured using a BIORAD Microplate reader at wavelength of 570 and 620 nm. The different absorbance values at 570 and 620 nm wavelength represented the direct correlation with number of viable cells per well. For anchorage-independent growth assay: soft agar plates were prepared in six-well plates with a bottom layer of 0.8 % Noble agar in serum-free DMEM 2 × 10^4^ cells mixed with 0.8 % Noble agar in 10 % fetal calf serum-supplemented DMEM were seeded as the top agar layer onto the agar plates. Colonies were visualized after six weeks culture by staining with 0.005 % crystal violet. Two wells were prepared for each treatment and the experiments were repeated twice.

### Western blot

Cells were lysed in a 500 μl lysis buffer (PBS, 0.5 % Triton X-100, 5 mM EDTA, and protease inhibitors) on ice for 30 min. The lysate was collected and cleared by centrifuging at 12,000 *g* for 20 min at 4 °C. Protein concentration of supernatants was determined by the Bradford’s method (Bio-Rad Laboratories protein assay, Hercules, CA, USA). 10 μg of the sample was separated by SDS-polyacrylamide gel electrophoresis and blotted as previously described [14]. Specific antibodies against AR, PSGR, Phosphorylated P70 S6 kinase, phosphorylated mTOR, and phosphorylated 4EBP1 were used for western blots (1:1000).

### Statistical analysis

Statistical analysis was performed using the Prism 5 statistical package from GraphPad Software, Inc. (La Jolla, CA). Data was analyzed and expressed as mean ± S.E. Overall survival (OS) was defined as the time from the date of diagnosis to the date of death or final clinical follow-up. The Kaplan-Meier method was used to estimate survival probabilities in patient subgroups. Significant difference of PSGR expression among/between groups was determined by one-way analysis of variance or Student’s *t*-test. A *p* value less than 0.05 was considered statistically significant.

## Results

### PSGR protein expression is significantly increased in PIN, but not in PCa

**Fig. 2:**
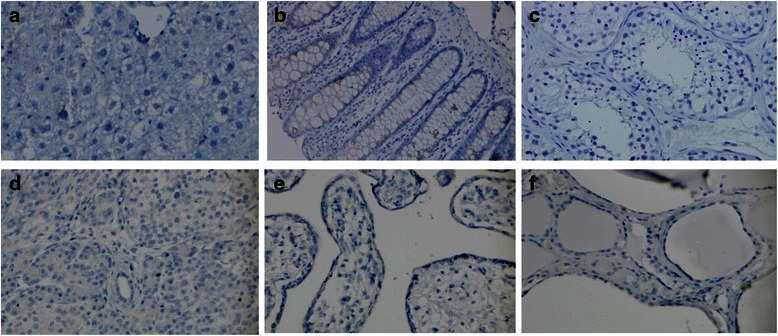
Expression of PSGR protein in other normal tissues by immunohistochemistry. PSGR staining in normal liver (**a**), colon (**b**), testes (**c**), pancreas (**d**), placenta (**e**) and thyroid (**f**) (original magnification x 20)

**Fig. 3:**
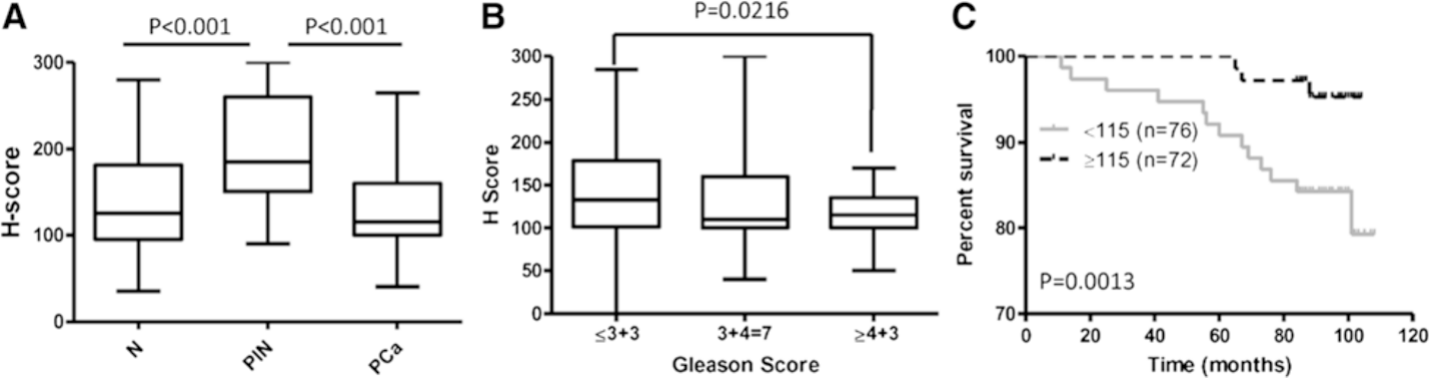
Expression levels of PSGR assessed by H-Score. **a** Distribution of H-Score of PSGR in adjacent normal prostate (N), PIN and PCa. **b** Association of PSGR protein expression with Gleason score. No statistically significant difference was observed between PCa and N. **c** Kaplan-Meier overall survival curves of PSGR expression

In our study, the distribution of PSGR in six types of normal human tissues and paired normal prostate, PIN, and PCa was tested with immunohistochemistry. Positive PSGR staining was located in the cytoplasm and cell membrane of normal prostate luminal epithelial cells (Fig. 1A), but not in normal liver, testes, colon, placenta or thyroid tissues (Fig. 2). Positive PSGR staining was also found in epithelial cells in PIN and PCa (Fig. 1A). Since the staining of PSGR could be patchy and staining intensity was variable in tissue samples (Fig. 1A), a H-score method was used to evaluate PSGR immunohistochemistry. A higher PSGR expression was found in PIN (H-score, 176.4 ± 5.12), compared to that in normal prostate (H-score, 132.6 ± 4.20, *P* < 0.001). Interestingly, the protein expression of PSGR decreased in PCa (H-score, 131.7 ± 4.37, *P* < 0.001) relative to PIN, which was close to that in normal prostate (Fig. 3a). Images in Fig. 1B clearly showed higher expression of PSGR in PIN compared to adjacent normal prostate or PCa in the same sections.

### The associations of PSGR expression with clinicopathological characteristics and patient overall survival

In our cohort of 150 patients, the median age at the time of PCa diagnosis was 61 years (range: 43 to 73 years). Survival information was available in 148 out of 150 patients. Expression of PSGR was not correlated with patient’s age, tumor stage, lymphovascular invasion or extraprostatic extension (Table 1). A lower PSGR protein expression was associated with higher Gleason pattern/score (Fig. 3b). The median H-score of PSGR in PCa was 115. Using the median score, the cohort was categorized into two groups, high expression of PSGR (H-score ≥115) and low expression of PSGR (H-score < 115). Kaplan-Meier analysis showed that low expression of PSGR was associated with poor OS (Fig. 3c).

**Table 1:** Associations of PSGR expression with clinicopathological characteristics of PCa

	PSGR	
	Mean ± SE	*P*
Age (years)		
≤ 61 (*n* = 72)	130.90 ± 6.72	0.95
> 61 (*n* = 78)	131.40 ± 6.03	
Tumor stage		
T2a (*n* = 26)	146.60 ± 11.90	0.33
T2b (*n* = 39)	127.61 ± 8.06	
T2c (*n* = 48)	124.21 ± 7.74	
T3+ (*n* = 37)	132.80 ± 7.52	
Lymphovascular invasion		
Present (*n* = 66)	125.30 ± 6.51	0.19
Absent (*n* = 84)	136.72 ± 5.87	
Extraprostatic extension		
Present (*n* = 65)	135.10 ± 6.99	0.83
Absent (*n* = 85)	132.90 ± 6.67	

### Activation of PSGR inhibits proliferation of C4-2 cells, but promotes cancer cell invasion

**Fig. 4:**
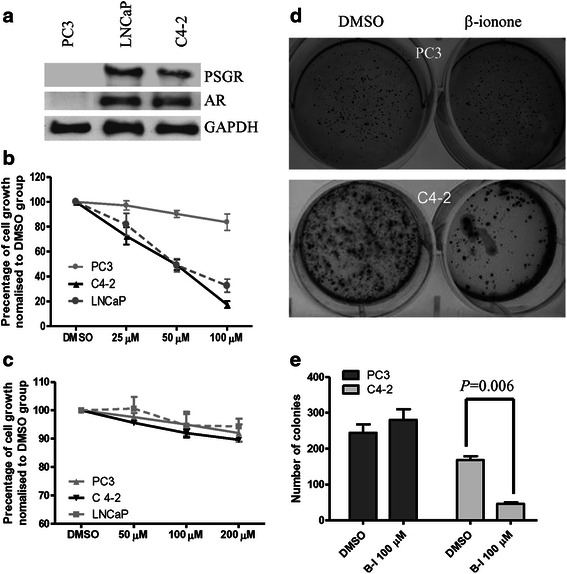
Activated PSGR by β-ionone inhibits C4-2 cell growth. **a** Western blot shows expression of PSGR protein in indicated PCa cell lines (*n* = 2). **b** MTT assay shows the effect of β-ionone on cell proliferation with indicated doses. C4-2, PC3 and LNCaP cells were treated for 3 days (*n* = 6). * indicated *p* < 0.05 compared to DMSO alone group. **c** Effect of α-ionone on C4-2, PC3 and LNCaP cell proliferation with indicated doses (*n* = 5). Cells were treated for 3 days. **d** Anchorage-Independent Growth Assay to evaluate the inhibitory effect of β-ionone on C4-2 cell growth. The image represents one of the three individual experiments. **e** Quantification data from anchorage-independent growth assay (*n* = 3)

**Fig. 5:**
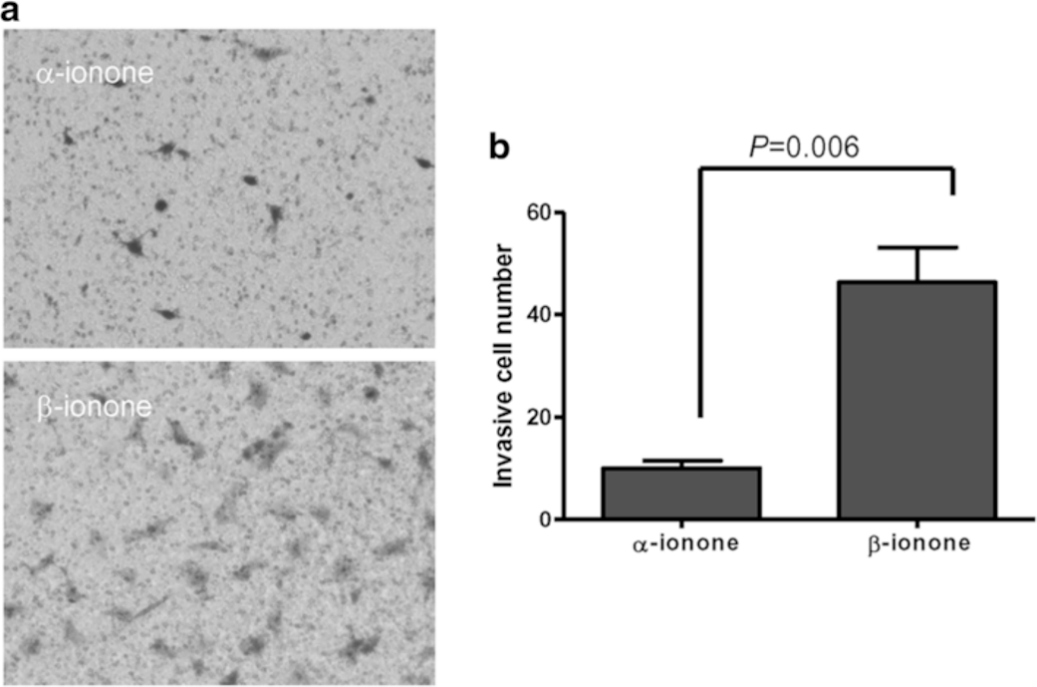
β-ionone reduced the invasive ability of C4-2 cells. **a** Representative images of invasive C4-2 cells stained with 0.1 % crystal violet (see detail Methods). **b** Quantification data from invasion experiments (*n* = 4)

We evaluated PSGR expression by Western Blot in several PCa cell lines including LNCaP, PC3 and C4-2 cells. The data indicated that C4-2 cells and LNCaP cells expressed PSGR, whereas PC3 cells were PSGR negative (Fig. 4a). PC3 cells were therefore adopted as a negative control in the study. We observed retarded proliferation of C4-2 cells after treatment with β-ionone, a PSGR agonist, following MTT and anchorage-independent growth assay (Fig. 4b, d and e). In contrast, PC3 cell proliferation was not altered by β-ionone treatment (Fig. 4b). In addition, we also tested the inhibitory effect of β-ionone on LNCaP cell growth in our study as a positive control. As previously reported [8], we found β-ionone retarded LNCaP cell growth in the study (Fig. 4b). To further identify the specific effect of β-ionone on PSGR in C4-2 cells, α-ionone, a substance structurally similar to β-ionone without the ability to activate PSGR [8], was employed as another negative control. As expected, α-ionone treatment did not alter the proliferation of C4-2 cells (Fig. 4c). Subsequently, we tested if activated PSGR affected cancer cell invasive ability. Our data showed β-ionone, but not α-ionone, promoted C4-2 cell invasion (Fig. 5a and b), which was consistent with recently published data from LNCaP cells [10].

### Phosphorylated p70 S6 kinase is decreased by activated PSGR

**Fig. 6:**
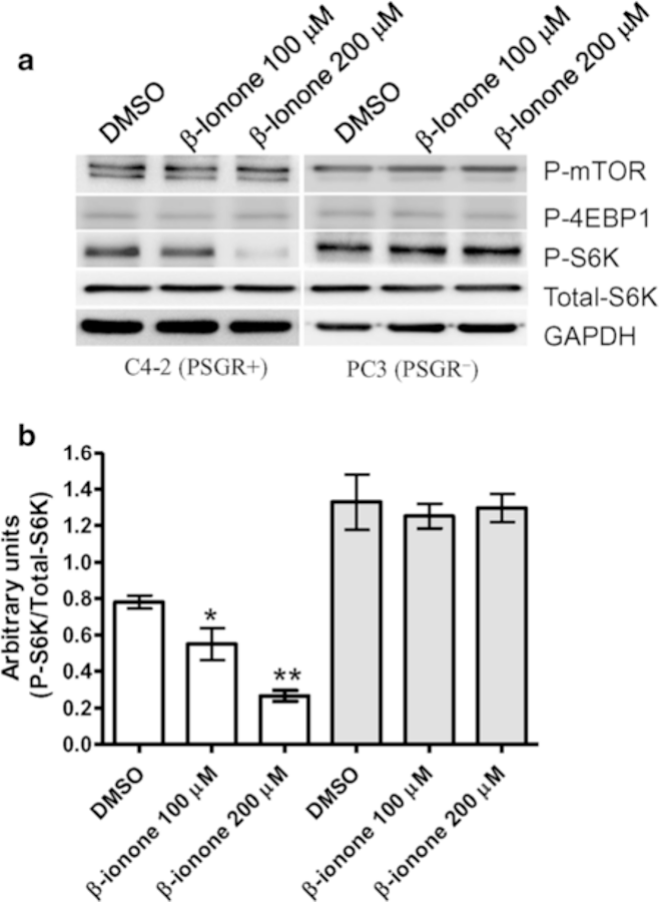
Phosphorylated status of mTOR, 4EBP1 and P70 S6 kinase. **a** C4-2 and PC3 PCa cells were treated by β-ionone for 3 days. The images of the blots represent one of the three individual experiments. P-mTOR, P-4EBP1 and P-S6K indicate phosphorylated mTOR, phosphorylated 4EBP1 and phosphorylated P70 S6 kinase, respectively. **b** Integrated optical density of each band measured by NIH Image J represents the expression of protein. Bar graphs show quantification of the expression level of P-S6K protein compared with the expression of GAPDH (Data are expressed as means ± SEM (*n* = 3). *p < 0.05 compared to DMSO group. **P < 0.05 compared to group treated with 100 μM β-ionone

A previous study with LNCaP cells revealed that PSGR directly activated Src kinase [9]. Src kinase is an important regulator for mTOR signaling in cancer [15]. Alterations in the mTOR pathway have been detected in PCa tissues in multiple studies, suggesting that this pathway plays an important role in the development and progression of PCa [16]. Inhibition of mTOR signaling has emerged as a potential therapeutic strategy for PCa [17, 18]. Data from this study indicated that activated PSGR retarded C4-2 cell growth and increased cell invasive ability. To evaluate if activated PSGR altered the mTOR signaling activity, we tested the activity of mTOR, and potential mTOR downstream molecules (p70 S6 kinase and 4EBP1) by Western Blot with specific phosphorylated antibodies. The data demonstrated that β-ionone stimulation did not alter mTOR and 4EBP1 activities in C4-2 cells (PSGR+) or PC3 cells (PSGR-). However, β-ionone treatment decreased p70 S6 kinase activity in dose dependent manner in C4-2 cells, but not in PC3 cells (Fig. 6a and b).

## Discussion

In this study, we evaluated the expression of PSGR protein in a large cohort of PCa patients using paired adjacent normal prostate, PIN, and PCa tissues. Our data not only confirmed PSGR specific expression in prostate luminal cells, but also revealed its dynamic pattern among normal prostate, PIN, and PCa. High expression of PSGR was seen in PIN, but not in PCa. Interestingly, low expression of PSGR protein in PCa was associated with high Gleason score and poor overall survival. This data suggested that PSGR may be a predictive marker for early PCa diagnosis and poor prognosis. In addition, our data from cell experiments indicated that PSGR might contribute to PCa progression by altering PCa cell growth and promoting cell invasion. P70 S6 kinase might be one of the important downstream molecules to mediate PSGR signaling. Notably, high grade PIN, a precursor of PCa, possesses the potential to develop to PCa. Further studies will be needed to identify the role of PSGR in the transformation of PIN to PCa.

Results from Quantitative-PCR and Northern blot have indicated that PSGR mRNA is relatively specifically expressed in prostate epithelial cells [2, 19, 20]. In the current study, PSGR protein expression was seen in normal prostate but not in testes, liver, colon, placenta, and thyroid tissues, providing more evidence for specific PSGR expression in prostate. Moreover, compared to non-cancerous controls, PSGR mRNA was significantly increased in PIN lesions [3]. In accordance with these findings, we showed highest PSGR protein expression in PIN compared to normal prostate and PCa, further suggesting that PSGR may play an important role in prostate cancer initiation. Notably, PSGR mRNA transcripts were shown to increase in PCa compared to that in non-cancerous tissues in several reports [2, 3, 20]. However, the data in this study showed a significant decrease of PSGR protein level in PCa compared to that in PIN. The differences of data may result from the following two reasons: 1) Previous studies evaluated the mRNA level of PSGR, whereas we studied PSGR protein expression. Evidence have delineated that about 60 % of variation in protein concentration was not able to be explained by knowing mRNA abundance [21]. 2) Previous studies isolated mRNA either from whole tissues or a small amount of desired prostate epithelial cells by laser micro-dissection [2, 3, 20]. It is possible that the tissues used for RNA isolation might have PIN mixed with PCa.

A recent study has demonstrated no apparent correlation between PSGR mRNA level and pathologic factors such as clinical stage, patient age, recurrent status, and serum PSA level before treatment [3]. The results from this study also showed no correlation of PSGR protein expression with patient age, clinical stage, lymphovascular invasion, or extraprostatic extension. However the data showed low PSGR expression was associated with high Gleason score, and poor OS. Our data suggested that PSGR protein expression might serve as a predictor for prognosis. Interestingly, detecting transcripts of PSGR in post-prostate massage urine seems to increase sensitivity and specificity of predicting PCa prognosis [7, 22]. Since PSGR expression is significantly increased in PIN and then decreased in PCa, we postulate that measuring PSGR protein expression dynamic change in post-prostate massage urine may be a reliable way to predict early PCa development.

There is limited information about PSGR signaling biological and pathological significance in prostate and PCa. Our data indicated that low PSGR protein level in PCa correlated with poor prognosis. Recent reports have demonstrated that activated PSGR with its selective agonist, β-ionone, inhibits LNCaP cell proliferation and increases its invasion ability [8, 10]. To explore the potential role of PSGR in tumor progression, we tested the effect of activated PSGR in C4-2 cells on cell growth and invasion. In agreement with the published results from LNCaP cells, β-ionone retarded C4-2 cell growth and increased cell invasion ability. This data further suggested that PSGR signaling might play an important role in PCa progression by disrupting cancer cell growth and promoting cell invasion. Manipulating PSGR activity might be a potential approach to treat PCa.

Several lines of evidence have suggested that MAPK, Src, and PI3K might be the downstream molecules for PSGR signaling [8–10]. PI3K and Src are important regulators for mTOR signaling. P70 S6 kinase and 4EBP1 are important downstream molecules of mTOR signaling transduction [23]. Studies have indicated that phosphorylation of p70 S6 kinase and 4EBP1 by mTOR plays an essential role in tumor growth and metastasis [24–26]. We hypothesized that activated PSGR might alter mTOR signaling activity. However, our data showed that activation of PSGR decreased p70 S6 kinase activity but did not change phosphorylated mTOR or 4EBP1 status in C4-2 cells. This data suggested that PSGR might regulate p70 S6 kinase activity in a manner independent of mTOR. Actually, G protein coupled receptor participating in regulating p70 S6 kinase activity in tumor cells has been reported in previous studies [27, 28].

## Conclusions

In summary, we demonstrated that PSGR protein was specifically expressed in normal prostate. Its expression was increased in PIN. Low expression of PSGR protein in PCa correlated with high Gleason score and poor overall survival. PSGR is not only a potential marker for predicting PCa initiation and prognosis, but also a potential target for PCa treatment.

## Abbreviations

AMACR: α-methylacyl-CoA racemase
ATCC: American type culture collection
BPH: Benign prostatic hyperplasia
MTT: 3-(4,5-dimethylthiazol-2-yl)-2,5- diphenyltetrazolium bromide
OS: Overall survival
PCa: Prostate cancer
PCA3: Prostate cancer gene 3
PIN: Prostatic intraepithelial neoplasia
PSA: Prostate specific antigen
PSGR: Prostate specific G protein coupled receptor
TMA: Tissue microarray
